# Barriers to childhood tuberculosis case detection and management in Cambodia: the perspectives of healthcare providers and caregivers

**DOI:** 10.1186/s12879-023-08044-y

**Published:** 2023-02-07

**Authors:** Yom An, Alvin Kuo Jing Teo, Chan Yuda Huot, Sivanna Tieng, Kim Eam Khun, Sok Heng Pheng, Chhenglay Leng, Serongkea Deng, Ngak Song, Daisuke Nonaka, Siyan Yi

**Affiliations:** 1 Sustaining Technical and Analytical Resources (STAR), The Public Health Institute (PHI), Phnom Penh, Cambodia; 2 School of Health Sciences, Faculty of Medicine, University of the Ryukyus, Okinawa, Japan; 3 School of Public Health, National Institute of Public Health, Phnom Penh, Cambodia; 4 Saw Swee Hock School of Public Health, National University of Singapore and National University Health System, Singapore, Singapore; 5 Faculty of Medicine and Health, University of Sydney, Sydney, NSW Australia; 6 National Center for Tuberculosis and Leprosy Control, Phnom Penh, Cambodia; 7 World Health Organization, Phnom Penh, Cambodia; 8 United States Agency for International Development, Phnom Penh, Cambodia; 9 KHANA Center for Population Health Research, Phnom Penh, Cambodia; 10 Center for Global Health Research, Touro University California, Vallejo, CA USA

**Keywords:** Tuberculosis, Childhood tuberculosis, Case detection, Barriers to services, Healthcare providers, Caregivers

## Abstract

**Background:**

Diagnosis and treatment of tuberculosis (TB) in children remain challenging, particularly in resource-limited settings. Healthcare providers and caregivers are critical in improving childhood TB screening and treatment. This study aimed to determine the barriers to childhood TB detection and management from the perspectives of healthcare providers and caregivers in Cambodia.

**Method:**

We conducted this qualitative study between November and December 2020. Data collection included in-depth interviews with 16 healthcare providers purposively selected from four operational districts and 28 caregivers of children with TB and children in close contact with bacteriologically confirmed pulmonary TB residing in the catchment areas of the selected health centers. Data were analyzed using thematic analyses.

**Results:**

Mean ages of healthcare providers and caregivers were 40.2 years (standard deviation [SD] 11.9) and 47.9 years (SD 14.6), respectively. Male was predominant among healthcare providers (93.8%). Three-fourths of caregivers were female, and 28.6% were grandparents. Inadequate TB staff, limited knowledge on childhood TB, poor collaboration among healthcare providers in different units on TB screening and management, limited quality of TB diagnostic tools, and interruption of supplies of childhood TB medicines due to maldistribution from higher levels to health facilities were the key barriers to childhood TB case detection and management. Caregivers reported transportation costs to and from health facilities, out-of-pocket expenditure, time-consuming, and no clear explanation from healthcare providers as barriers to childhood TB care-seeking. Aging caregivers with poor physical conditions, lack of collaboration from caregivers, ignorance of healthcare provider's advice, and parent movement were also identified as barriers to childhood TB case detection and management.

**Conclusions:**

The national TB program should further invest in staff development for TB, scale-up appropriate TB diagnostic tools and ensure its functionalities, such as rapid molecular diagnostic systems and X-ray machines, and strengthen childhood TB drug management at all levels. These may include drug forecasting, precise drug distribution and monitoring mechanism, and increasing community awareness about TB to increase community engagement.

**Supplementary Information:**

The online version contains supplementary material available at 10.1186/s12879-023-08044-y.

## Background

With an estimated 10 million new cases in 2019, tuberculosis (TB) remains a major global health problem [1]. South-East Asia accounted for 43% of the total cases [2]. Childhood TB contributed about 12% of the total estimated TB cases [1], ranging from 2 to 7% in high-income countries to 15% to 20% in low-income countries [3]. Despite significant progress, TB case detection remains a big challenge. Based on the World Health Organization (WHO), approximately 35% of the global TB cases of all ages are missed (under-diagnosis and under-reporting). This proportion is up to 55% in children [4].

Recently, WHO excluded Cambodia from the global list of high TB burden countries from 2021 to 2025 [5] as the country has reached the 2020 milestone of reducing 20% of TB incidence [6]. Despite this remarkable achievement, TB incidence in the country remains high. In 2020, WHO estimated that 46,000 Cambodians or 274 per 100,000 population became sick with TB, while only 29,136 TB cases (63.3% of the estimated incidence cases) were reported in the same year [7]. Childhood TB cases are also under-reported in Cambodia. In 2020, only 6,557 childhood TB were reported to the national TB program [7], equivalent to only 68.3% of the total of 9,600 childhood TB cases estimated by The Global TB Report 2021.

Diagnosis and treatment of tuberculosis (TB) in children remain challenging, particularly in resource-limited settings. In Cambodia, TB diagnosis delay in adults with TB is associated with living in rural areas, presenting symptoms (cough, haemoptysis, and night sweat), using private services or self-medication, and TB-related self-stigma [8]. Another study reported that refusal of TB diagnosis, indirect costs, and fear of TB medicines’ side effects were the key barriers to acceptance of TB treatment [9]. A recent study published in 2021 identified key barriers to accessing TB services for populations at high risk of TB, including limited knowledge of TB, lack of TB awareness, financial burden, time constraints, and gender-specific vulnerabilities [10]. The study also found some barriers from the provider side, such as the inconsistent implementation of policies and guidelines and limited resources for TB [10].

Only a few studies have investigated factors associated with and identified barriers to childhood TB screening and management in low- and middle-income countries. In Peru, incorrect TB drug dosage, staff workload, and concern about Isoniazid resistance due to preventive therapy were the critical barriers to treating childhood TB [11]. Limited TB knowledge of healthcare providers was a barrier to diagnosis, treatment, and prevention of childhood TB in several studies [4, 12, 13]. In Cambodia, it was reported that only 72.5% of clinicians working at TB wards could identify five out of seven main childhood TB screening criteria, and their knowledge may not be well applied to practices [12].

Previous studies have identified several barriers to TB detection and management in different contexts. A recent quantitative study in Cambodia focused on clinicians’ knowledge, availability of diagnostic tools for childhood TB at referral hospitals, symptoms in children for which caregivers sought treatment, and how clinicians conducted clinical examinations for childhood TB diagnosis based on caregivers’ observation [12]. Given the lack of in-depth understanding of this issue, the present study sought to understand barriers to childhood TB case detection and management in Cambodia from the perspective of healthcare providers and caregivers.

## Methods

### Study design and setting

We conducted a qualitative study between November and December 2020 in four operational districts of two provinces and the capital of Phnom Penh in Cambodia. The selection of respondents from the four operational districts was based on their knowledge score on childhood TB, reported in a quantitative survey. Cambodia’s public health system is divided into the national level (central), provincial level, and operational district level [14]. Operational districts provide management and technical support to referral hospitals, health centers, and health posts in providing health services, including TB care [14].

### Study population

We purposively selected 16 healthcare providers with high knowledge scores about childhood TB, including four operational district TB supervisors, four TB clinicians, four TB nurses from the referral hospitals, and four TB nurses from health centers. The participants' childhood TB knowledge scores were determined based on the answers to the questions related to causes of TB, transmission routes, signs and symptoms, characteristics of lymph nodes that implied TB, diagnostic criteria for childhood TB, and TB prevention, such as how to prevent TB transmission, the use of TB preventive treatment (TPT), the target group for TPT, medicines used for TPT, contraindication of TPT, and possible side effects of TPT. The detailed knowledge score has been reported separately [15]. We recruited a convenience sample of 28 caregivers of children under 15 years old with previous or undergoing TB treatment and children in close contact with people with bacteriologically-confirmed pulmonary TB. Caregivers of children with close contact with TB index cases had experiences of accompanying the children under their care to undergo TB screening. Based on Cambodia’s national guidelines on latent TB infection, people with close contact with TB index cases, including children, are required to undergo TB screening. Caregivers who resided in the catchment area of selected health facilities and were 18 years old or older were eligible for the study. Healthcare providers pre-identified eligible caregivers, and the research team selected caregivers based on their availability and geographical convenience. Caregivers were persons who provided care to the children during sickness and accompanied them to seek health care, TB diagnosis, and treatment, not limited to parents and immediate family members. The recruitment of caregivers was done under the facilitation of local healthcare providers.

### Data collection tools

We developed semi-structured interview guides (see Additional file 1) in consultations with the Cambodia Committee for TB Research. The interview guides included questions on socio-demographic characteristics of respondents, barriers to childhood TB case detection and management, and barriers to TPT implementation among children. The findings on the barriers to TPT implementation have been reported in a separate manuscript. We used inductive approach to generate the themes on the barriers to childhood TB case detection and management. We pre-tested the tools before the actual data collection to ensure that the language used was contextually understandable, validate the content, estimate the interview time, and ensure data quality and the familiarity of the data collection teams with the tools.

### Data collection procedure

Face-to-face in-depth interviews (IDIs) were conducted in Khmer by well-trained, experienced Cambodian interviewers with medical backgrounds. Prior arrangements were made with the participants. The interviews with healthcare providers were done at their working places and with caregivers at their houses. All interviews were conducted in a private room or place with audio recordings and field notes taken. It took between 30 to 40 min to complete the interview. During data collection, meetings were held at the end of each day to discuss the interview processes, including the interview content. The confirmation of data saturation was done during the data collection period.

### Data management and analyses

Recorded data were transcribed verbatim and then translated into English. Key themes were verified against Khmer transcription, and data were analyzed using thematic analyses. First, we developed a codebook of themes based on the semi-structured interview guides. Then, we coded the transcripts manually through discussions between two researchers. We performed inductive thematic analysis and also considered emerging themes and sub-themes.

### Ethics approval and consent to participate

This study was approved by the National Ethics Committee for Health Research (NECHR) (ref. 234/NECHR) in Cambodia and the Ethics Review Committee of the World Health Organization Western Pacific Regional Office (WPRO-ERC 2020.8.CAM.3.STB). All participants provided written informed consent for the study. All methods were carried out in accordance with the Declaration of Helsinki. The objectives, procedures, risks, and benefits of participating in this study were explained to all participants. A written informed consent form was read or verbally explained to each participant, and written consent was obtained from all participants before the interviews. All data, including name, address, and personal information, were treated anonymously and strictly handled confidentially. Each participant received a compensation gift valued at about US$ 1 after the interview.

## Results

### Socio-demographic characteristics

The characteristics of respondents are presented in Table 1. The mean age of healthcare providers was 40.2 years (SD 11. 9), and 93.8% were male. Among caregivers, the mean age was 47.9 years (SD 14.6), and 75.0% were female. Eight out of twenty-eight caregivers were grandparents, one-fourth had no formal education, and 60.7% were farmers. Three of out 28 or (10.71%) caregivers cared for more than one child.

**Table 1:** Demographic characteristics of IDI participants

Demographic characteristics	Number (%)
Healthcare providers (N = 16)	
Age in years, mean (SD)	40.2 (SD 11. 9)
Sex, male	15 (93.8)
Working place	
Operational district	4 (25.0)
Referral hospital	8 (50.0)
Health center	4 (25.0)
Role	
TB supervisor	4 (25.0)
Clinician in charge of TB at referral hospitals	4 (25.0)
Nurse in charge of TB at referral hospitals	4 (25.0)
Nurse in charge of TB at a health center	4 (25.0)
Caregivers (N = 28)	
Age in years, mean (SD)	47.9 (SD 14.6)
Sex, female	21 (75.0)
Relationship of caregivers with children	
Parent	20 (71.4)
Grandparent	8 (28.6)
Caregivers	
Caregivers of children currently or previously on TB treatment	9 (32.14)
Caregivers of children currently or previously on TPT	11 (39.29)
Caregivers who refused TPT for their eligible children	8 (28.57)
Education	
No formal education	7 (25.0)
Primary school (1–6 years)	8 (28.6)
Secondary school (7–9 years)	10 (35.8)
High school or higher (≥ 10 years)	3 (10.7)
Main occupation	
Farmer	17 (60.7)
Seller	5 (17.9)
Government or private sector staff	2 (7.1)
Other	4 (14.3)

IDI: in-depth interview, SD: standard deviation; TB: tuberculosis, TPT: TB preventive treatment

### Lack of human resources and collaboration

Several healthcare providers reported a lack of human resources for TB as a barrier to providing TB services. The lack of human resources included a shortage of staff in charge of TB, limited staff capacity in childhood TB, and the lack of collaboration within the health facilities in providing TB services for children.

*"But what is lacking for him [healthcare provider] is knowledge [on childhood TB], I want to say that the staff is new and has been trained late. Some staff took the role for about two to three years but had not yet received any training (both adult and childhood TB). So, this is the obstacle” (Operational district TB supervisor, IDI-1, female, 35).*

*"Our human resources are still lacking. From the beginning, I had not been involved with TB work. However, the hospital director transferred me to handle TB. By the time, TB training [childhood TB] was limited … our skills remained limited." (Doctor working at referral hospital, IDI-16, male, 35).*

*"The problem is TB screening. When the patients first come to the health centers, they go to the Outpatient Department or Triage section. The doctors don't help us screen the patients for TB unless he/she is a doctor in charge of TB. So, we lose the cases even we try hard to do it. Our aim is to screen TB for everyone with symptoms or with presumptive TB." (Operational district TB supervisor, IDI-7, male, 31).*

Caregivers also perceived that the unavailability of staff in charge of TB at health facilities was a barrier to getting TB services. The shortage of service providers results in a long waiting time and multiple visits to get the services.

*"It is also difficult … When my daughter reached the hospital, one doctor did not come to see her; other doctors did not come to see her. My daughter traveled three to four times to meet doctors successfully. The healthcare providers said the other doctors did not come when my daughter met one doctor. We traveled a long way from here to the hospital." (Caregiver IDI-4, female, 49, with an 11-year-old child on TPT).*

*“I waited from 9 AM until 12 PM to get the result. And they told me to get the medicines (for my kid) at the health center.” (Caregiver IDI-2, male, 40 with children aged 8 years old, 6 years old and 2 years old on TPT).*

### Lack of TB diagnostic tools

Almost all healthcare provider respondents reported a lack of TB diagnostic tools such as good quality chest X-ray machines, limited access to rapid molecular diagnostic systems such as the GeneXpert® MTB/RIF, low or irregular supplies of X-ray films, poor quality microscopes, and lack of other support materials for making TB diagnosis.

*"…. because the X-ray machine is too old, we cannot take many pictures. So, when many children came, they had to wait for days to get X-ray." (Operational district TB supervisor, IDI-1, female, 35).*

*"We want to request GeneXpert*® *because it is easy for TB diagnosis. In this hospital, there is no film for X-rays. We bought X-ray films using the hospital’s budget. On the other hand there was a problem with microscopes in the hospital during this last period. The other day, we asked someone to check, and we also put a request to the upper level." (Operational district TB supervisor, IDI-2, male, 39).*

### Low involvement from the community

Several challenges in providing TB services were also due to the low community participation and collaboration in TB case detection and management. This low involvement could be due to caregivers' movement or busyness, ignorance of providers' advice, poor understanding of TB in the community, or low-risk perception of TB.

*“My challenge is from patients' families. They are busy. They work at the factories. They don't have enough time to take care of their children and cannot come to the hospital regularly. Sometimes, health center staff explain them, and volunteers bring them to the hospital and back, but they don't come. This happens because of the patients themselves…..sometimes, we refer them …….. they just say yes, yes, but in fact, they don't go". (Health center nurse in charge of TB, IDI-3, male, 31).*

*"Although we try hard to encourage parents to bring their kids for TB screening, only some of them listen to our advice and bring their kids to see us. … The most common reason is their economic status; their parents are too poor and have to immigrate to work in other countries. Thus, it isn’t easy to bring the kids to the hospital as appointed". (Doctor working at a referral hospital in charge of TB, IDI-9, male, 36).*

*“Another problem is related to their thought. Caregivers think that TB is not a serious issue. Therefore, they didn’t bring their kids to us for TB work up”. (Health center nurse in charge of TB, IDI-12, male, 28).*

### Interruption of drug supplies or short shelf-life medicines for childhood TB

Providers reported a short period of childhood TB medicine unavailability due to poor or late distribution from higher levels. In addition, some childhood TB medicines had a short shelf-life. These interruptions of supplies posed challenges in providing treatment for childhood TB.

*"For childhood TB treatment, the medicines [for childhood TB] were not available last semester. Medicines [for childhood TB] at the national TB program were unavailable, and central medical store (CMS) also did not supply because the medicines were out of stock." (Operational district TB supervisor, IDI-1, female, 35).*

*“During this last period, we faced problems with expired medicines for childhood TB. We requested the national TB program, but the delivery was late, so we requested it from the province. We then received some medicines [for childhood TB] from the provincial health department, but the medicines were almost expired … now, many medicines are in stock.” (Operational district TB supervisor, IDI-2, male, 39).*

Caregivers similarly reported that the unavailability of childhood TB medicines was a barrier when accessing TB services.

*"My husband was the one who went to get the medicines [for my son]. I only stayed at home. When he went to get the medicines, they ran out, so he did not get the medicines immediately. We got the medicines after the next few days." (Caregiver IDI-9, female, 32 with a 9-year-old child on TB treatment).*

*“Only one concern, when I go to get medicines… Normally, the doctor said TB medicines were out of stock, so he could not give me on time, so I am afraid that ….. since the doctor told me that taking TB medicines need to be well adhered, …. my kid may take medicines on and off (one day take, one day not), so he may need to restart the treatment.” (Caregiver IDI-1, female, 25 with a 4-year-old child on TB treatment).*

### Aging caregivers

In this study, eight out of 28 caregivers interviewed were grandparents. Both healthcare providers and caregivers reported that caregivers’ old age was a barrier in childhood TB case detection and management. One grandparent who took care of his grandchild with TB reported that it was difficult to bring his grandchild to the health center to get TB services since he was too old. Another caregiver, a grandmother of a child with TB, reported forgetting everything as she was too old. Healthcare providers reported similar constraint.

*“…but it is difficult for me to travel there [health center] because I am old.” (caregiver IDI-2, male, 62 with a 5 years old child on TB treatment)*

*“The main challenge for childhood TB is identifying them as their parents move around for work, and it is not easy to refer them [children] to TB services because their grandparents are old and cannot come. In this village, almost 90% of the children are taken care of by elderly people when their parents go to work.” (Operational district TB supervisor, IDI-1, female, 35).*

### Other barriers

#### Out-of-pocket expenditures

One caregiver reported having been asked to pay for the sputum test and other services within the hospital.

*" I did not go with my daughter, I forgot how much she paid maybe ….hmm…. like more than 10,000 riels (about 2.5 USD) or less than 10,000 riels. We then had to pay for medical services at other places. When she was diagnosed with TB, we still pay them (healthcare providers)." (Caregiver IDI-4, female, 49 with an 11-year-old child on TPT).*

#### Transportastion costs

Although TB services were provided free of charge, one healthcare provider reported that some caregivers did not have money to travel to receive TB services.

*“As I know, the problem is not different as I mentioned previously, it is difficult for such children to travel to receive TB services because they live with old grandparents. Moreover, they need to travel for a long and don’t have money to travel here.” (Operational district TB supervisor, IDI-1, female, 35).*

Caregivers also raised transportation fees as a challenge when bringing their children to TB services.

*“They [doctors] did not charge the money, but I needed to spend on transportation.” (caregiver IDI-4, female, 45 with a 8-year-old child on TB treatment)*

#### Issues in communication with healthcare providers

Unclear instruction on how to take TB medicines was reported by a caregiver as a barrier, resulting in the discontinuation of TB treatment for her son.

*“He [my son] missed taking (TB) medicines for two months because the first time he got the medicines, the doctor did not tell him to come back and get more [medicines] when he ran out [of medicines]. He only took the medicines for one month, and he felt fine, so he did not go to get more medicines.” (Caregiver IDI-5, female, 40 refused TPT for her a 2-year-old child).*

### Suggestions to increase childhood TB detection

Several healthcare providers suggested that increasing community awareness and strengthening TB screening at triage, outpatient, and inpatient departments were key strategies for improving childhood TB detection. Improving contact investigation, performing active case detection, strengthening human resources, and providing incentive support were also suggested by a few healthcare providers to improve childhood TB case detection.

*"We can go to the villages to provide health education. If a child is a presumptive TB case, ask him/her to get TB services at health centers or referral hospitals as soon as possible." (Operational district TB supervisor IDI-2, male, 39).*

*"….we have to provide health education to parents and caregivers. We must provide education to the local authorities if they have time. The chiefs of communes and village health support groups can participate and provide health education." (Health center nurse, IDI-5, male, 62).*

*"……. expanding the screening. As I said, doctors at the triage departments should ask if the patients have coughs because we want to capture 100% of presumptive TB cases. So, I want to refer the (presumptive TB) cases from the triage directly to the TB doctor. I want to screen as much as possible, even the patient with flu symptoms”. (Operational district TB supervisor, IDI-7, male, 31).*

*“….. first, I want my human resources to be stable. If the human resources are not stable as nowdays, then I do not know how to strengthen them because the staff always changes. I want to have TB staff who could stay longer”. (Operational district TB supervisor, IDI-7, male, 31).*

## Discussion

This study assessed the barriers to childhood TB case detection and management from the perspective of healthcare providers and caregivers of children with TB. The studies depicted multiple barriers in childhood TB case detection and management. The lack of human resources for TB services, including either staff shortage or limited staff capacity to screen, diagnose, and treat childhood TB, was identified. These findings are similar to those from several studies in other countries [16–20]. Diagnosing childhood TB is often more complicated than in adults. Therefore, comprehensive training on childhood TB is required to strengthen the providers’ capacity. The national TB program should further invest in childhood TB training, targeting healthcare providers who are newly involved with the TB program.

Poor collaboration among providers from different services within health facilities, mainly at triage, to screen children for TB was identified in this study. This situation is a context-specific barrier in Cambodia. Through the joint program review of the national TB program in 2012 and 2019, internal referral linkages to facilitate early detection of TB cases were identified as a weak point and needed to be strengthened, such as hospital engagement to screen TB within each hospital department and unit, especially diabetes, paediatrics, and HIV clinics and wards [21, 22]. This finding suggested that more engagement in TB screening, awareness-raising, and staff capacity building must be strengthened among TB providers and other providers involved in TB detection within the same health facilities. Screening for childhood TB should be done for all children who come to seek healthcare in different services within the health facilities. Based on WHO, in settings where the TB prevalence in the general population is 100/100,000 population or higher, systematic screening for active TB should be considered among people seeking health care [22].

The hospital engagement model has been proved to increase TB case notification. In Pakistan, systematic engagement of hospital administration and all specialist doctors, staff training, and regular facility-based review meetings were associated with a 35% increase in childhood TB case detection during the same period [23]. In Cambodia, from 2015 to 2017, FHI-360, through the Challenge TB project, piloted a project called Hospital Linkage in five provinces by identifying 7816 TB cases [24]. In 2018, this model was expanded to 10 hospitals under the support of the Global Fund to Fight AIDS, Tuberculosis, and Malaria [25]. A joint TB program review in 2019 also recommended strengthening TB management in public hospitals through internal coordination to detect and notify TB early and improve TB case management [21]. In 2022, this model was implemented in referral hospitals in 70 operational districts under the Global Fund’s support and in 10 operational districts under the support of the United States Agency for International Development (USAID). The model should be adapted and expanded to other health facilities to strengthen TB screening and management.

All providers reported the lack of quality TB diagnostic tools and supplies of relevant consumables as a limitation to childhood TB detection. This challenge is common, especially in resource-limited settings. In Peru, a lack of adequately functioning radiograph machines was identified as a barrier to childhood TB case detection, and this problem was solved by referring patients to private facilities [16]. A previous study in Cambodia also depicted the low availability of proper childhood TB diagnostic tools [12]. The joint TB program review in 2019 identified limited access to chest X-ray machines and routine specimen collection for TB diagnosis TB in children [21]. These could delay the diagnosis of childhood TB, and caregivers might bring their children to other services in the private sector.

In Cambodia, childhood TB was high, representing 22.5% of the total 29,136 notified TB cases in 2020 [7], and almost all notified childhood TB cases in 2020 were clinically diagnosed [26]. This situation may lead to empirical and inappropriate treatment and a low treatment success rate [27]. Improving the quality of childhood TB diagnosis through investment in diagnostic tools such as GeneXpert® MTB/RIF and digital X-ray machines should be prioritized. Since early February 2021, the national TB program in Cambodia has deployed GeneXpert® Ultra as an initial test for diagnosing TB among the general population, including children in 30 operational districts. The program will be further expanded to other locations in the following years. This intervention is in line with a rapid recommendation recently released by WHO aiming to inform national TB programs and other stakeholders about the critical implications of the latest evidence on the use of specific molecular assays as initial diagnostic tests of pulmonary and extrapulmonary TB and rifampicin-resistant TB in adults and children [28].

Recently, WHO released a new guideline for managing TB in children and adolescents [29]. The guideline recommends that, for children with signs and symptoms of pulmonary TB, in addition to sputum samples, nasopharyngeal or gastric aspirate or stool samples should be collected and tested by Xpert Ultra for detecting TB cases and rifampicin resistance [29]. The national TB program may consider this new recommendation to add on the existing childhood TB diagnostic tools in the country.

Low community involvement was perceived as a barrier to childhood TB case detection and management. These challenges may be specific to the Cambodian context where children are looked after by their grandparents, who would hesitate to accept TB treatment for their grandchildren or decide on behalf of their parents on their grandchildren’s health. Patients' movement and the busyness of caregivers were also perceived as barriers to providing childhood TB services. In addition, caregivers’ ignorance of the seriousness of TB and doctors' advice by the community may result in poor management of childhood TB. In Peru, parental ignorance about TB was the reason for late or undiagnosed TB in children [16]. Poor understanding of TB among the community was also perceived as a barrier. This finding is consistent with studies in Indonesia and Bangladesh [30–32]. In a recent study, caregivers who were male and ≥ 45 years old and those with no formal education and poor knowledge of the importance of adherence to TB treatment were the predictors significantly associated with non-adherence to the TB treatment [33].

The availability and quality of TB medicines are essential to ensure optimal TB treatment outcomes. In this study, healthcare providers and caregivers reported the unavailability of childhood TB medicines and the availability of medicines with short shelf-life as barriers to providing childhood TB treatment. In Papua New Guinea, interruption of supplies was reported to cause people with TB to be given a partial supply of medicines, and interruptions in TB treatment could occur when TB medicines were unavailable [34]. The shortage of medicines was identified as a factor associated with poor adherence to anti-TB treatment in India [35]. In Cambodia, free anti-TB medicines were provided to patients nationwide. The central medical store is responsible for distributing TB medicines to operational districts that further distribute them to health facilities where TB patients come to collect TB medicines. Childhood TB drug unavailability at lower-level health facilities could be due to poor TB drug management, although the drugs may be available at the central level. The national TB program should strengthen the childhood TB drug supply chain mechanism to ensure uninterrupted supplies, such as appropriate drug forecasting or strengthening the coordination between staff at the TB program and pharmacies at each level. Temporary solutions that have been practiced in some health facilities, such mobilization of childhood TB drugs between nearby health facilities, should be encouraged to ensure the continuation of childhood TB treatment.

A healthcare provider reported transportation costs as barriers to accessing TB services. The transportation costs may interrupt access to TB services and delay childhood TB diagnosis and management, resulting in poor TB treatment outcomes and a financial burden to the families. In central and western Nepal, lack of transportation was a barrier to TB service utilization [36]. In Ethiopia, transportation costs resulted in TB treatment interruptions [37] and posed a significant challenge in seeking TB treatment in India [38].

While TB services have been provided free of charge at public health facilities in Cambodia [39], a caregiver reported that her daughter spent some money while seeking TB care at public health facilities, although it was unclear what the money was for. Out-of-pocket expenditures have been reported while seeking care at public health facilities in Cambodia, but not specifically for TB services [40, 41]. This finding is similar to studies in other countries where TB diagnosis and treatment were provided for free, but patients still experienced out-of-pocket expenditures [42, 43]. This may impose a significant financial burden on the family and lead to catastrophic costs.

It was time-consuming to access TB service. This could be due to staff shortage and could lead to a substantial TB diagnosis and treatment delay and increase the opportunity of disease transmission. A similar challenge was identified in other studies. Due to the nature of health infrastructures in sub-Saharan Africa, over 90% of patients spent an average of four visits before their TB diseases were diagnosed [44]. In Botswana, caregivers visit health facilities repeatedly due to the delay in TB test results, leading to a delay in the initiation of TB treatment for children [45]. To address this challenge, decentralizing childhood TB diagnosis and management, such as further allocating human resources and diagnostic tools for childhood TB, may be needed.

Two of the three grandparents who were the caregivers of children with TB reported physical difficulty and memory loss. Generally, grandparents are the older adults with poor physical and mental health and may even face financial constraints [46]. This group of caregivers should receive special attention and support to improve childhood TB case detection, management, and other health outcomes. For instance, in this study setting, a grandfather, who was too old to bring his grandchild to the health center, received support from the village chief to facilitate TB medicine collection from the health center.

### Study limitations

This study has some limitations. First, reporting bias may occur since data were collected through face-to-face interviews. Second, this study collected the personal perspectives of healthcare providers and caregivers in Cambodia. Some identified barriers are context-specific for Cambodia or the selected study sites; hence they may not be directly generalized to other settings. Third, there was a gender imbalance among the respondents. Most caregiver participants (75%) were female and may experience fewer barriers to accessing childhood TB services than male caregivers. In Cambodia, men are usually the family’s breadwinners and may work far from home or health facilities resulting in difficulty accessing TB services during operating hours. Finally, recruiting caregivers by local healthcare providers may lead to selection bias. To reduce reporting bias, the interviewers reiterated the study’s objectives and the implications of the findings and reassured the participants regarding privacy and that whatever they said would not have any negative repercussions on them.

## Conclusions

The findings suggest that the national TB program should further invest in human resources for TB. This could be done by a national strategy for human resources for TB, focusing on staff capacity building on childhood TB, providing staff motivation, and allocating more staff in charge of TB in health facilities. These strategies align with WHO’s recommendations in the roadmap toward ending TB in children and adolescents [4]. Expanding the hospital linkage model to other referral hospitals should also be done to improve the collaboration among healthcare providers in different services within the referral hospitals to screen and refer children with presumptive TB for diagnostic workups. TB clinics need to be equipped with adequate TB diagnostic tools for childhood TB diagnoses, such as GeneXpert® MTB/RIF, X-ray machines, and X-ray films, with regular maintenance to ensure the functionality of the tools.

Further efforts from the national TB program and other stakeholders are needed to strengthen Cambodia’s free TB service policy to reach “zero catastrophic cost for affected families due to TB” as stated in the national strategic plan 2021–2030 [39]. Childhood TB drug management at all levels should be strengthened, such as drug forecasting, a precise mechanism for drug distribution, and drug monitoring, to avoid supply interruptions. Furthermore, interventions to address context-specific challenges are needed to maximize childhood TB case detection and management in Cambodia. These may include increasing community awareness of TB and special support to aging caregivers to improve childhood TB case detection and management in Cambodia.

## Supplementary Information

**Additional file 1.** Interview guide for In-Depth Interviews (IDIs).

## Data Availability

Data and materials are available upon request from Dr. Yom An (Email: anyomniph@gmail.com).
