# Supplementary material for: Barriers to childhood tuberculosis case detection and management in Cambodia: the perspectives of healthcare providers and caregivers
Source: BMC Infect Dis. 2023 Feb 7;23:80. doi: 10.1186/s12879-023-08044-y (PMC9903406; doi:10.1186/s12879-023-08044-y)
Supplement: Supplementary file 1 — Additional file 1. Interview guide for In-Depth Interviews (IDIs). [file 12879_2023_8044_MOESM1_ESM.docx]

**Interview guide for In-Depth Interviews (IDIs)**

| **Part 1: IDI with healthcare providers** | | |
| --- | --- | --- |
| **Demographic information** | | |
| Q1 | Sex  Male 1  Female 2 | |
| Q2 | Age (in years): _________________ Years |  |
|  |  |  |
| Q3 | Working place | |
|  | OD: (specify: …...........................................) 1  RH: (specify: …...........................................) 2    HC: (specify: …...........................................) 3 | |
| Q4 | Role  TB OD supervisor 1    Clinician working at TB ward at RH 2    Nurse working at TB ward at RH 3    Nurse working at HC 4    Other: …............................. 5 | |
| **Barriers to childhood TB case detection** | | |
| Q5 | What are the barriers in providing TB service in general? (human resource, workload, infrastructure, facilities, ….....) | |
| Q6 | What are the challenges in identifying presumptive childhood TB in your coverage area? (screening process? workload? Capacity? Motivation? Guidance from central level…....) | |
| Q7 | What are the challenges in diagnosing childhood TB in your OD? (Capacity? Diagnostic tools? Motivation? Guidance from central level? ….......) | |
| Q8 | If a presumptive TB child need to be referred for TB diagnostic workup, what are the challenges in referring? (distance? Road condition? Support? No transportation means? | |
| Q9 | If a child is diagnosed with TB, what are the challenges or barriers in providing TB treatment? (Capacity? Availability of TB drug, time, …... ) | |
| Q10 | How to increase childhood TB case detection in your coverage area? (think of strategy, specific interventions, motivation support, strengthen referral system ….. ) | |
| **Barriers to TPT implementation among children** | | |
| Q11 | What do you know about TPT? What is TPT? | |
| Q12 | How do you think about TPT? (effective for TB prevention? Safe? Should TPT be widely implemented? ….............) | |
| Q13 | Do you think TPT is important? Why? | |
| Q14 | What are the barriers or challenges in providing TPT? (think of knowledge of providers, guidance from upper level, lack of risk perception by caretakers, availability of drug, …...) | |
| Q15 | What should be done (your recommendation) to improve TPT implementation? | |
| **END** | | |

| **Part 2: IDIs with caregivers** | |
| --- | --- |
| **Demographic information** | |
| Q1 | Your current address  Village: …..............................; Commune: …..............................;  District: ….............................., Province: …................................. |
|  |  |
| Q2 | Sex (of caregivers)    Male 1    Female 2 |
|  |  |
|  |  |
| Q3 | Age of caregiver: _________________ Years |
|  |  |
| Q4 | Relationship to children  Parent 1    Grandparent 2    Other: …................................ 3 |
|  |  |
|  |  |
|  |  |
| Q5 | Your kid  is confirmed and treated with TB 1    is receiving TPT 2    is eligible for TPT, but I don’t allow to take TPT 3 |
|  |  |
|  |  |
|  |  |
| Q6 | Age of his/her children: _________________ Years |
|  |  |
| Q7 | Relationship of his/her child with index case:  Living in the same house 1    Close contact 2    Neighbour 3    None of above 4 |
|  |  |
|  |  |
|  |  |
|  |  |
| Q8 | Education of caregiver  Never go to school 1    Primary school 2    Secondary school 3    High school 4    Post high school 5 |
|  |  |
|  |  |
|  |  |
|  |  |
|  |  |
| Q9 | Main occupation of caregiver  Farmer 1    Seller 2    Business 3    Government official 4    Retired 5  Private sector 6    Other: …................................ 7 |
|  |  |
|  |  |
|  |  |
|  |  |
|  |  |
|  |  |
|  |  |
| **Barriers to childhood TB case detection** | |
| Q10 | What is tuberculosis? |
| Q11 | How do you know that a people has TB? |
| Q12 | What are the barriers in accessing TB service? (think of distance? Service providers? Cost? Quality of service? Stigma? …. ) |
| Q13 | If your kid is suspected of having TB, what are the challenges/barriers in getting TB diagnosis? |
| Q14 | If your kid diagnosed with TB, what are the challenges or barriers in getting TB treatment? |
| **Barriers to TPT implementation among children** | |
| Q15 | What do you know about TB prevention? |
| Q16 | Have you ever heart about TPT? If yes, who tell you? What is it for? Is it safe to take? If not safe why?  If no, interviewer briefly tell him/her about TPT. |
| Q17 | How do you think about TPT? (effectiveness, cost, …. ) |
| Q18 | If your kid is eligible for TPT, would you accept TPT for your kid? If not, why? |
| **END** | |
